# Comprehensive opportunistic osteoporosis screening with AI: retrospective development and prospective validation of dual-site BMD prediction from a single radiograph

**DOI:** 10.3389/fendo.2026.1906679

**Published:** 2026-07-23

**Authors:** Hsuan-Yin Lin, Jyh-Wen Chai, Qingzong Tseng, Cheng-Wei Lin, Kuan-Jung Pan, Kai-Wei Liu, Ya-Chu Shih

**Affiliations:** 1 Department of Medical Imaging, Taichung Veterans General Hospital, Taichung, Taiwan; 2 School of Medicine, College of Medicine, National Yang Ming Chiao Tung University, Taipei, Taiwan; 3 Department of Medical Research, Taichung Veterans General Hospital, Taichung, Taiwan; 4 Alpha Intelligence Manifolds, New Taipei City, Taiwan; 5 Master of Computational Data Science, Carnegie Mellon University, Pittsburgh, PA, United States

**Keywords:** AI, deep learning, dual-site BMD prediction, opportunistic screening, osteoporosis

## Abstract

**Purpose:**

To develop and validate an AI algorithm that enables dual-site (spine and hip) BMD assessment for opportunistic osteoporosis screening using a single kidney-ureter-bladder (KUB) radiograph.

**Materials and methods:**

In this institutional review board approved prospective study, we developed the SHield pipeline for opportunistic osteoporosis screening. From an initial dataset of 15,175 KUB images, a final cohort of 4,436 patients (mean age 69.0 ± 12.2 years) was included for model development after exclusions for suboptimal quality or incomplete region of interests. The pipeline analyzes both the spine and hip regions on a single KUB to predict site-specific BMD values. Finally, it integrates these dual-site predictions to determine the lowest T-score, mimicking the standard clinical DXA reporting protocol which evaluates both the lumbar spine and proximal femur.

**Results:**

On the internal test set (628 patients), the hip and spine AI models demonstrated Pearson correlation coefficients of 0.887 and 0.921 and RMSEs of 0.057 and 0.062, respectively, compared to DXA-measured BMD, achieving AUCs of 0.894 and 0.956 for predicting T-scores ≤-2.5. In a subsequent prospective feasibility study (51 patients), we employed a conservative detection threshold of T_m_-score ≤-2.8 to minimize false positives and unnecessary referrals. The final AI pipeline achieved a Pearson correlation of 0.959, an AUC of 0.969, 100% PPV, 100% specificity, and 83.8% NPV.

**Conclusion:**

This paper presents the first AI pipeline that enables gold-standard-aligned, dual-site BMD estimation and opportunistic osteoporosis screening from a single KUB radiograph. Validated prospectively, its high accuracy and specificity confirm its potential to significantly increase early detection rates without overwhelming clinical workflows.

## Highlights

- This study presents the first radiogrammetry-based AI model for estimating BMD and screening for osteoporosis that leverages a single, preexisting KUB radiograph to perform a dual-site evaluation of both the spine and hip. On the internal test set, these AI models demonstrated high accuracy in BMD estimation (Pearson correlation coefficients, 0.887−0.921) and strong discriminatory ability for osteoporosis (area under the receiver operating characteristic curve [AUCs], 0.894−0.956).
- In a real-world prospective feasibility study, the final AI pipeline achieved excellent performance for opportunistic screening, including an AUC of 0.969, 100% positive predictive value [PPV], and 100% specificity at a conservative T_m_-score threshold of ≤−2.8.

## Introduction

Osteoporosis, a chronic metabolic bone disease, is characterized by reduced bone mineral density (BMD) and a consequent decline in bone strength (1, 2). This condition substantially increases the risk of fragility fractures, which are associated with disability, mortality (3), diminished quality of life, and a significant financial burden on both families and healthcare systems (4). Due to global population aging and increased life expectancy, osteoporosis is rapidly becoming a worldwide epidemic. Despite the potential for prevention and management with early intervention, osteoporosis remains underdiagnosed and undertreated among at-risk populations (5, 6). This discrepancy is primarily attributed to limited public awareness of osteoporosis, coupled with restricted accessibility to Dual-energy X-ray Absorptiometry (DXA) examinations.

Opportunistic osteoporosis screening has emerged as a potential research field in recent years (7, 8). This approach utilizes medical images originally acquired for other diagnostic purposes, offering a cost-effective method to potentially improve the identification of osteoporosis. Radiogrammetry provides a relatively inexpensive method for estimating BMD from conventional radiographs by analyzing morphological parameters (9, 10). Prior AI studies focused on opportunistic BMD prediction have utilized chest, lumbar spine, and hip images (11–18); however, these radiographic views are not routinely obtained in standard health examinations. Moreover, chest X-rays lack the necessary data to evaluate hip BMD and associated fracture risk – a critical factor in morbidity and mortality among elderly individuals with osteoporosis (19). In contrast, kidney-ureter-bladder (KUB) radiographs are routinely acquired and provide valuable information on both lumbar spine and hip, aligning with the primary diagnostic sites for gold-standard DXA.

The purpose of this study was to develop and validate an AI algorithm that estimates dual-site BMD from a single, routine KUB radiograph for opportunistic osteoporosis screening.

## Materials and methods

### Study design and overview

This study employed a two-phased design: a retrospective model training phase followed by a prospective feasibility test phase. The Institutional Review Board (IRB number: SF23547B) and the Development Center for Biotechnology (DCB-IRB-112001) granted ethical approval for this research. Informed consent was waived for the retrospective model training phase, as the data utilized was de-identified. For the prospective feasibility phase of the study, informed consent was obtained from all participants prior to the analysis of study records.

### Data acquisition

#### Model training phase

**Figure 1:**
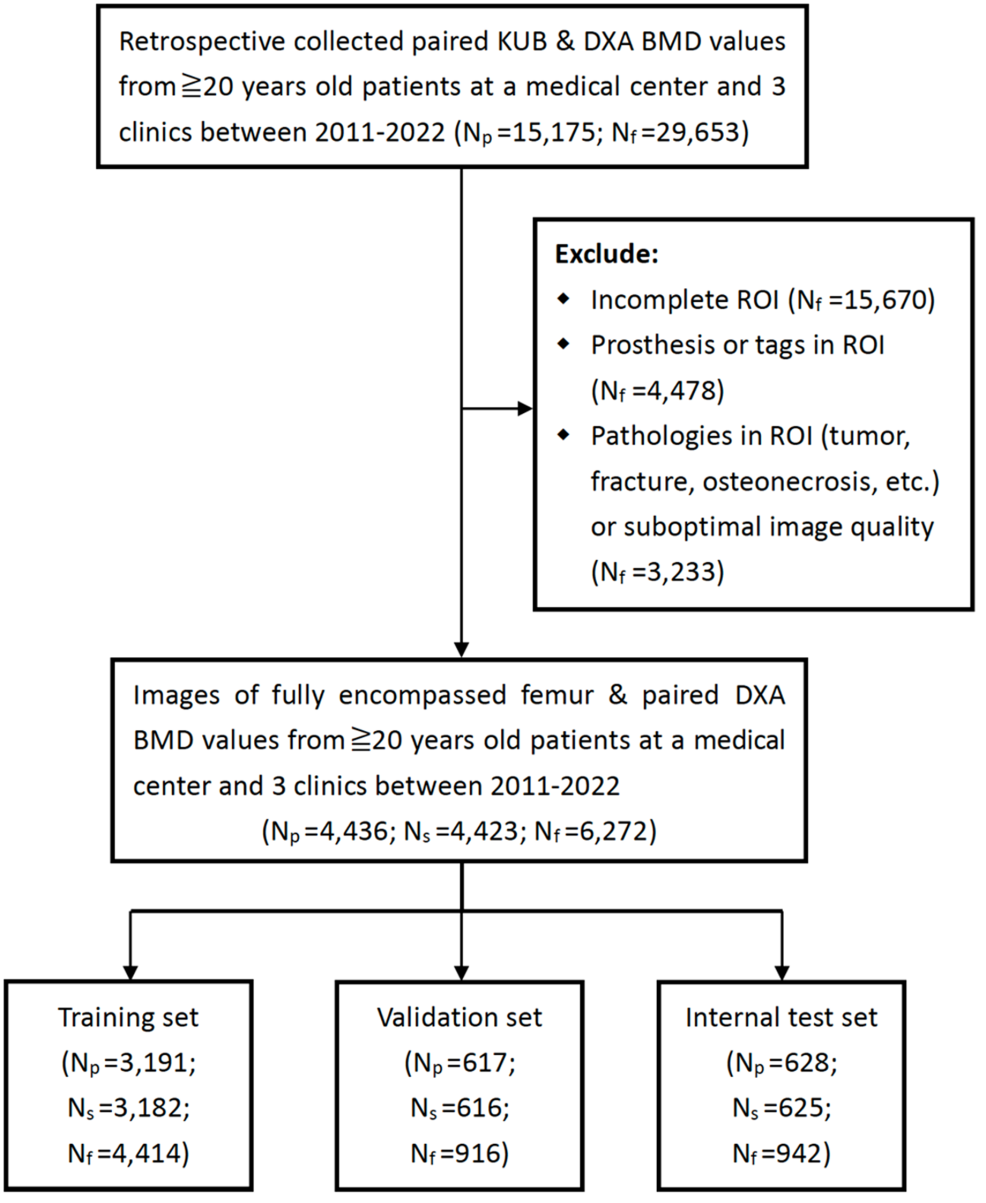
Flow diagram of data inclusion and allocation for model development. Np, number of patients; Nf, number of femurs; Ns, number of spines; KUB, Kidney-Ureter-Bladder; DXA, dual-energy X-ray absorptiometry; BMD, bone mineral density; ROI, regions of interest.

For the model training phase, we retrospectively collected 15,175 de-identified paired DXA BMD values and KUB radiographs, obtained from patients over 20 years of age at a medical center and three local clinics between October, 2011, and November, 2022. Importantly, all three clinics and the medical center shared a unified PACS system and utilized the identical GE Lunar iDXA machine (GE Healthcare, Chicago, Illinois, USA) for scanning, thereby eliminating cross-institutional hardware variance. Standard site-specific DXA scans independently evaluating the lumbar spine and proximal femur were performed. The temporal interval between the KUB radiograph and the DXA scan was strictly limited to ≤6 months to ensure temporal alignment and preserve ground truth validity. After excluding images with incomplete regions of interest (ROIs), prostheses or tags, pathologies in the femur or spine ROIs or suboptimal image quality, 6,272 paired KUB images and DXA BMD values from 4,436 patients were enrolled for model development. We split the dataset into a training set, validation set, and internal test set (**Figure 1**). To prevent data leakage, dataset splitting was performed strictly at the patient level; repeated longitudinal scans from the same patient were exclusively assigned to the training set. This patient-level allocation strategy inherently resulted in a higher prevalence of osteoporosis in the training set (46.7%), as patients with multiple scans tended to be older and under active osteoporosis surveillance. Conversely, the validation and internal test sets were maintained to reflect the natural disease prevalence of the screening population (approximately 28-30%), aligning with established global and Taiwanese local epidemiological estimates (2, 4, 6). This final dataset was then used to train the two distinct deep learning models that form the core of the SHield pipeline: one dedicated to estimating BMD from the lumbar spine and another for the hip.

#### Feasibility test phase

For the feasibility test phase of the study, we employed stratified sampling to recruit 100 participants aged 50 years or older with a KUB radiograph performed within the preceding year. For a better representation of the real-world population distribution, stratification was conducted to align the sex and age distribution of the study sample with the demographic profile of the Taiwanese population in 2022 (**Supplementary Table 1**). Each participant’s pre-existing KUB radiograph was processed by the SHield pipeline, which generate dual-site BMD and T-score predictions for both the spine and hip, ultimately selecting the lowest T-(or Z-)score as the final diagnostic output. Subsequently, participants underwent a standard DXA BMD measurement, these DXA values served as the ground truth for evaluating the model’s real-world performance.

### SHield pipeline development

#### Radiographs preprocessing

Initially, each input KUB radiograph was padded to a 1:1 aspect ratio and intensity-normalized. Subsequently, to focus on the clinically relevant anatomy for BMD prediction, objection detection models were trained to automatically localize the spine and hip joints. The spinal region of interest (ROI) was defined as rectangular regions spanning from T12 to L5. The hip joint ROI was a rectangular area encompassing the femoral head, proximal femoral shaft, and both the greater and lesser trochanters (LTR) bilaterally. This design ensures the ROIs effectively encompass the key anatomical regions assessed in standard DXA scans.

These detection models utilized a pre-trained YOLOv5 (20) model fine-tuned on radiographs of the hip, pelvis, KUB, and lumbar spine. Non-Maximum Suppression (NMS) (21) was also performed as a post-processing step to identify the hip regions with the highest confidence scores in the outputs.

#### Feature-driven informed network for data refinement

For more accurate feature extraction, we employed radiogrammetric analysis and a hip landmark model to assess anatomical feature significance within the hip ROI. This HRFormer (22)-based model localized hip landmarks within ROIs extracted from the detection model, representing them as graphs.

**Figure 2:**
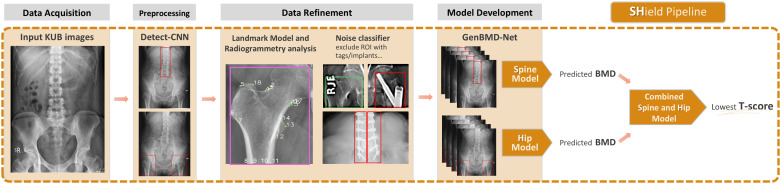
Schematic of the SHield pipeline, an end-to-end workflow for dual-site BMD prediction from a single KUB radiograph. The workflow includes four main stages: Data acquisition, Radiographs preprocessing, Data refinement, and Model development. The SHield pipeline integrates the predicted BMD from the Spine and Hip Models to determine the lowest BMD value and the corresponding lowest T-score for opportunistic osteoporosis screening. KUB, Kidney-Ureter-Bladder; CNN, convolutional neural network; ROI, regions of interest; BMD, bone mineral density.

The hip landmark model detected 18 distinct anatomical landmarks within the hip ROI, including seven landmarks on the femoral head and neck, four on the greater trochanter, three on the LTR, and four on the shaft (**Figure 2**). Corresponding heatmap confidence scores indicated the prominence of each anatomical feature (23). Ultimately, landmarks on the LTR were the highest-scoring anatomical features within the hip ROI.

To verify that input hip ROIs for BMD model training fully contained the LTR, the highest-scoring feature, we trained an automated classification model using a RegNetY-16GF (24) backbone. Finally, only ROIs with a complete LTR and without medical conditions—such as implants, foreign bodies, or pathologies—were used for downstream BMD prediction model training. While anatomical landmark localization was specifically applied to the hip ROI to capture the complex geometry of the proximal femur, the lumbar spine model extracted features directly from the bounding box ROI, as vertebral bodies present more uniform morphological patterns on AP radiographs (**Figure 2**).

#### Spine and hip model: deep learning-based model development

These selected ROIs served as input for training the BMD prediction models. Specifically, this dual-site prediction task was addressed using two independent deep learning models—one dedicated to the spine and one to the hip. Both models independently integrated a RegNetY-16GF (24) backbone with a series of fully connected regression layers to output predicted BMD values.

The Mean Squared Error (MSE) was the primary loss function. A Mutual-Information Regularization with Oracle (MIRO) loss function was further incorporated to address the feature space disparity between this trained and the oracle models, which used the ImageNet RegNetY-16GF to enhance this model’s generalizability (25).

#### The SHield pipeline: an end-to-end dual-site BMD prediction workflow

The entire end-to-end workflow, termed the SHield pipeline, integrates the Spine and Hip models into a single tool for opportunistic screening (**Figure 2**). From a single KUB input, the pipeline generates dual-site BMD predictions and their corresponding T-scores, reporting the lowest one as the final output. This methodology was designed to align with the gold-standard DXA protocol, which utilizes the minimum value from two key anatomical sites for clinical diagnosis. Our models were implemented with PyTorch, version 1.5 (https://pytorch.org).

### Statistical analysis

To evaluate the AI model’s performance in opportunistic osteoporosis screening using hip BMD, we employed several statistical methods. Patient characteristics across the training, validation, and internal test sets were described using means and standard deviations for continuous variables (age, BMD T-scores) and counts with percentages for categorical variables (sex, BMD categories). Differences in continuous variables between groups were assessed using the Mann-Whitney U test, while categorical variable distributions were compared using Chi-squared or Fisher’s exact tests.

Diagnostic performance of the AI model was quantified using several metrics, each reported with 95% confidence interval (CI). These metrics included positive predictive value (PPV), negative predictive value (NPV), sensitivity, specificity, root mean squared error (RMSE) and mean absolute error (MAE). The overall discriminatory ability of the model was further assessed using the area under the receiver operating characteristic curve (AUC). For diagnostic performance metrics reaching 100% (e.g., PPV and Specificity), 95% CIs were calculated using the Clopper-Pearson exact method. This provides appropriate asymmetric bounds for binomial proportions, yielding a more conservative and reliable estimate of uncertainty. The strength and direction of the linear relationship between model predictions and DXA measurements were evaluated using Pearson’s correlation coefficient (*r*). Model performance was evaluated against various osteoporosis definitions derived from DXA T-scores at the femoral neck, total hip, and a combination of hip and lumbar spine. T-scores were calculated by comparing BMD values to the NHANES young White female reference data, complying with the International Society for Clinical Densitometry (ISCD) and Taiwanese clinical guidelines, which mandate a uniform reference across all ethnicities to standardize fracture risk assessment (26, 27). Subgroup analyses were also performed across different T_m_-score thresholds to examine their influence on diagnostic accuracy.

A *P* value below 0.05 was considered indicative of statistical significance. All statistical analyses were conducted using the R software environment (version 3.0.0; R Foundation for Statistical Computing, Vienna, Austria).

## Results

### Study sample characteristics

For model training and pipeline development, we divided the study sample into three datasets: a training set comprising 3,182 fully encompassed spines and 4,414 femurs images from 3,191 patients), a validation set (616 spines and 916 femurs from 617 patients), and an internal test set (625 spines and 942 femurs from 628 patients). The training group consisted of 2,734 women (mean age 69.4 ± 12.2 years) with the following BMD distribution: normal (8.4%), osteopenia (44.9%), osteoporosis (46.7%). The validation group included 530 women (mean age 68.3 ± 11.5 years) with a BMD distribution of normal (12.3%), osteopenia (57.9%), and osteoporosis (29.8%). The internal test set comprised 537 women (mean age 68.0 ± 12.2 years) with 11.0% normal BMD, 60.2% osteopenia, and 28.8% osteoporosis, detailed patient characteristics for hip and spine model development are shown in **Table 1**.

**Table 1:** Patient characteristics stratified by dataset in BMD AI model development*.

Characteristics	Training set	Validation set	Internal test set	P value
No. of patients	3191	617	628	
No. of spines	3182	616	625	
No. of femurs	4414	916	942	
Age (year, mean ± SD)	69.4 ± 12.2	68.3 ± 11.5	68.0 ± 12.2	0.024
Sex				0.6
Women	2,734 (85.7%)	530 (85.9%)	537 (84.5%)	
Men	457 (14.3%)	87 (14.1%)	91 (14.5%)	
BMI (kg/m2, mean ± SD)	25.03 ± 3.73	24.98 ± 3.41	25.09 ± 3.28	<0.001
Femur Neck BMD (g/cm2, mean ± SD)	0.713 ± 0.132	0.750 ± 0.128	0.751 ± 0.119	<0.001
Total Hip BMD (g/cm2, mean ± SD)	0.798 ± 0.147	0.840 ± 0.146	0.843 ± 0.139	<0.001
Femur Neck T-score (mean ± SD)	-2.3 ± 0.9	-2.1 ± 0.9	-2.1 ± 0.9	<0.001
Total Hip T-score (mean ± SD)	-1.7 ± 1.2	-1.3 ± 1.2	-1.3 ± 1.1	<0.001
BMD categories				<0.001
Normal	267 (8.4%)	76 (12.3%)	69 (11.0%)	
Osteopenia	1433 (44.9%)	357 (57.9%)	378 (60.2%)	
Osteoporosis	1491 (46.7%)	184 (29.8%)	181(28.8%)	

*Values are numbers (percentages) unless indicated otherwise.

BMD, bone mineral density; AI, artificial intelligence.

For prospective validation, a feasibility study of 100 participants assessed real-world applicability. Following the SHield pipeline’s automated filtering, the final analysis cohorts included 99 participants for the spine model, 51 for the hip model, and 51 for the complete dual-site evaluation.

### Performance of BMD AI model

#### Performance in the internal test set

**Figure 3:**
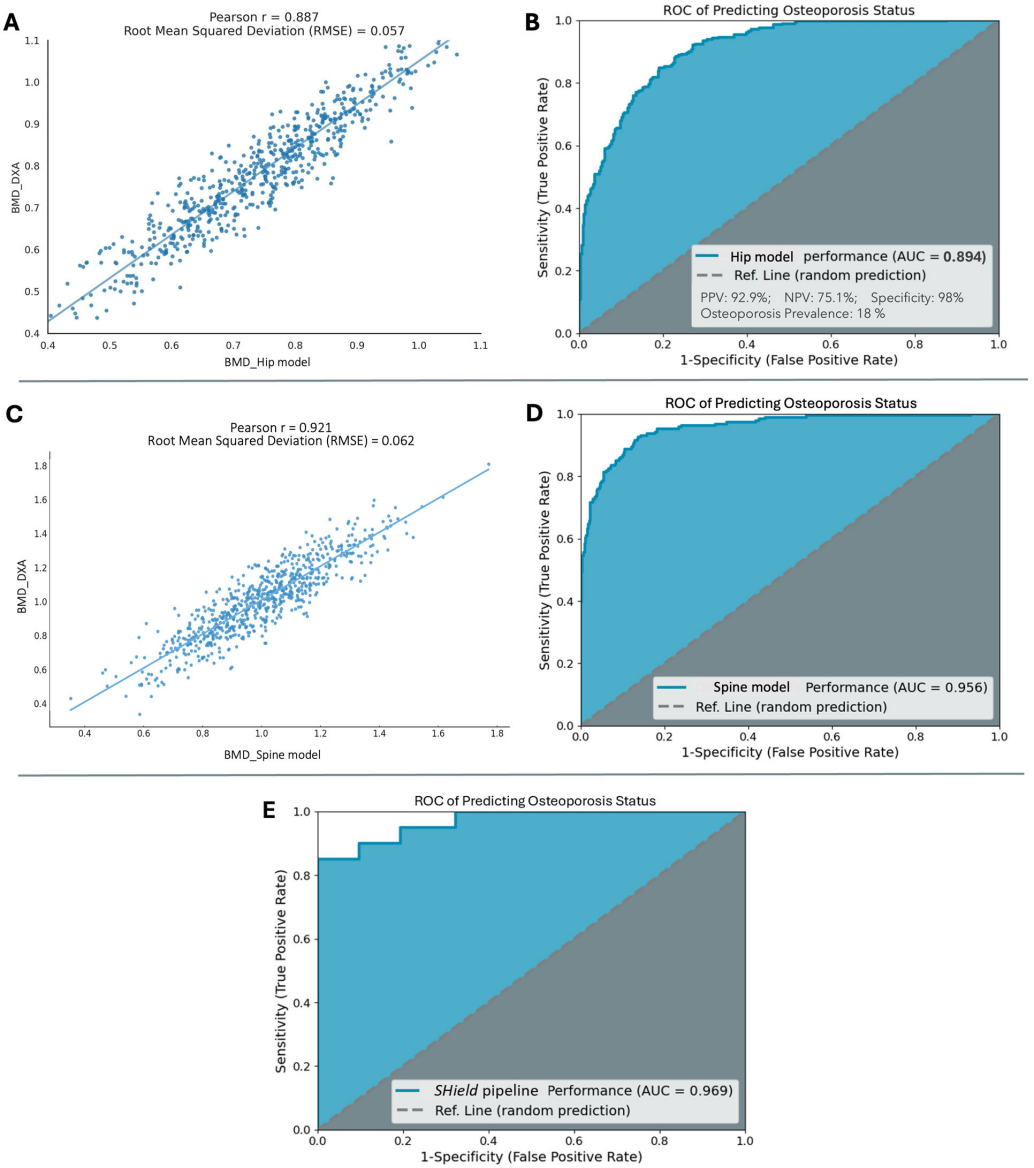
Performance of the BMD AI Model for BMD estimation and osteoporosis prediction. **(A–D)** illustrate the model’s performance on the internal test set (n=628) and **(E)** on the prospective feasibility study (n=51). **(A, C)** show scatter plots of model-predicted BMD versus DXA-measured BMD for the Hip Model and Spine Model, respectively, with Pearson correlation coefficient (r) and RMSE indicating accuracy. **(B, D)** display the corresponding ROC curves, assessing diagnostic performance for osteoporosis prediction using AUC. **(E)** displays the ROC curve of *SHield* pipeline on the prospective feasibility study. BMD, bone mineral density; DXA, dual-energy X-ray absorptiometry; RMSE, root mean square error; ROC, receiver operating characteristic; AUC, area under the receiver operating characteristic curve; PPV, positive predictive value; NPV, negative predictive value.

Regarding BMD estimation accuracy, the Hip Model achieved an *r* of 0.887 (95% CI: 0.870-0.902) and an RMSE of 0.057 g/cm² (0.055-0.060) in comparison with DXA-measured BMD. The Spine Model achieved an *r* of 0.921 (95% CI: 0.903-0.933) and an RMSE of 0.062 g/cm² (0.058-0.068) (**Table 2**; **Figures 3A, C**).

**Table 2:** Performance* of the BMD AI model in the internal test set: hip and spine model and subgroup analysis by T_m_-Score threshold.

MODEL	AUC (95%CI)	PPV (%, 95%CI)	NPV (%, 95%CI)	Sensitivity (%, 95%CI)	Specificity (%, 95%CI)	RMSE (95%CI)	MAE (95%CI)	Pearson’ r (95%CI)	OP prevalence^‡^ (%)
Hip^#^	0.894 (0.868- 0.917)	79.1 (73.2- 84.6)	80.7 (77.1- 84.3)	63.5 (57.3-70.0)	90.1 (87.1-92.8)	0.057 (0.055- 0.060)	0.046 (0.044- 0.048)	0.887 (0.870-0.902)	29.8
Spine^#^	0.956 (0.938- 0.970)	86.6 (81.0- 91.4)	91.9 (89.5- 94.0)	75.9 (69.5-81.5)	95.9 (94.1-97.4)	0.062 (0.058- 0.068)	0.048 (0.045- 0.051)	0.921 (0.903-0.933)	26.0
Modified Hip T_m_-score Threshold									
-2.6	0.894 (0.868- 0.917)	84.9 (78.9- 90.1)	78.2 (74.3- 81.8)	55.4 (48.7-61.7)	94.2 (91.7-96.3)				24.2
-2.7	0.894 (0.868- 0.917)	90.3 (85.2- 95.1)	77.3 (73.6- 81.1)	51.9 (45.0-58.7)	96.7 (94.9-98.4)				21.3
-2.8	0.894 (0.868- 0.917)	92.9 (87.7- 97.3)	75.1 (71.4- 78.9)	45.1 (38.6-51.2)	98.0 (96.5-99.2)				18.0

*Osteoporosis ground truth was determined by DXA T-scores at hip and lumbar spine.

^#^A T_m_-score ≤-2.5 was used as default detection threshold for osteoporosis (T_m_-score: T-scores derived from model-predicted.

BMD values).

‡ OP prevalence was determined by applying the detection threshold to the T_m_-score.

BMD, bone mineral density, AI, artificial intelligence, AUC, area under the receiver operating characteristic curve, PPV, positive predictive value, NPV, negative predictive value, RMSE, root mean squared error, MAE, mean absolute error, OP, osteoporosis, DXA, dual-energy X-ray absorptiometry.

We evaluated the models’ performance in predicting osteoporosis against a ground truth defined by the lowest T-score of ≤−2.5 from the L1-L4 spine, femoral neck or total hip, as measured by DXA. The Hip Model and Spine Model achieved AUCs of 0.894 (95% CI: 0.868−0.917) and 0.956 (95% CI: 0.938−0.970), respectively. (**Table 2**; **Figures 3B, D**). Using a T_m_-score (T-scores derived from model-predicted BMD values) threshold of ≤-2.5, the Hip Model achieved a specificity of 90.1% (95% CI: 87.1-92.8), and a sensitivity of 63.5% (57.3-70.0) in predicting osteoporosis. The corresponding PPV and NPV were 79.1% (95% CI: 73.2-84.6) and 80.7% (77.1-84.3), respectively, with a predicted osteoporosis prevalence of 29.8%. In opportunistic screening, minimizing false positives is clinically imperative to avoid unnecessary anxiety and resource burden. Therefore, we evaluated the sensitivity-specificity trade-offs across different Tm-score thresholds. While the default ≤-2.5 threshold yielded a balanced sensitivity and specificity, shifting to a more conservative threshold of ≤-2.8 was deliberately selected to prioritize downstream clinical utility. This adjustment significantly increased PPV to 92.9% (87.7-97.3) and specificity to 98.0% (96.5-99.2), making it highly suitable for screening purposes at the acceptable expense of lower sensitivity (**Table 2**).

#### Performance in the prospective feasibility study

Both the individual Spine and Hip Models maintained high estimation accuracy in the prospective feasibility study. The Spine Model achieved an *r* of 0.959 (95% CI: 0.943−0.973) and an RMSE of 0.070 g/cm² (0.060−0.080); the Hip Model yielded a correlation of 0.894 (95% CI: 0.840−0.929) and an RMSE of 0.056 g/cm² (0.048−0.064). The SHield pipeline, integrating these models for end-to-end dual-site BMD prediction from a single KUB, achieved an *r* of 0.959 (95% CI: 0.946−0.970) and an RMSE of 0.060 g/cm² (0.053−0.067), indicating a highly accurate and robust linear relationship with the ground truth (**Table 3**).

**Table 3:** Performance* of the BMD AI model in the feasibility study: hip, spine & *SHield* pipeline^#^.

AUC (95% CI)	PPV (%, 95%CI)	NPV (%, 95%CI)	Sensitivity (%, 95%CI)	Specificity (%, 95%CI)	RMSE (95% CI)	MAE (95% CI)	Pearson’ r (95% CI)	OP prevalence^‡^ (%)
Hip model								
0.890 (0.787- 0.966)	100.0 (59.0- 100.0)^§^	70.5 (56.5- 83.7)	35.0 (14.3- 57.9)	100.0 (88.8- 100.0)^§^	0.056 (0.048- 0.064)	0.044 (0.036- 0.051)	0.894 (0.840- 0.929)	39.2
Spine model								
0.940 (0.893- 0.980)	100.0 (59.0- 100.0)^§^	79.1 (70.2- 87.2)	43.8 (26.7- 60.7)	100.0 (90.0- 100.0)^§^	0.070 (0.060- 0.080)	0.057 (0.050- 0.065)	0.959 (0.943- 0.973)	32.0
*SHield* pipeline^#^								
0.969 (0.924- 1.000)	100.0 (76.8- 100.0)^§^	83.8 (72.7- 94.6)	70.0 (50.0- 89.5)	100.0 (88.8- 100.0)^§^	0.060 (0.053- 0.067)	0.048 (0.042- 0.054)	0.959 (0.946- 0.970)	39.2

* OP ground truth used lowest T-score derived from DXA true BMD values at hip and spine to determine OP classification.

# *SHield* pipeline used lowest T-score derived from hip and spine model-predicted BMD values to determine OP classification.

‡ OP prevalence was determined by applying the conservative detection threshold (T_m_-score ≤-2.8) to the T-scores derived from model-predicted BMD values.

§ For diagnostic performance metrics reaching 100%, 95% confidence intervals were calculated using the Clopper-Pearson exact method.

BMD, bone mineral density; AI, artificial intelligence; OP; osteoporosis; DXA, dual-energy X-ray absorptiometry; AUC, area under the receiver operating characteristic curve; PPV, positive predictive value; NPV, negative predictive value; RMSE, root mean squared error; MAE, mean absolute error.

**Figure 4:**
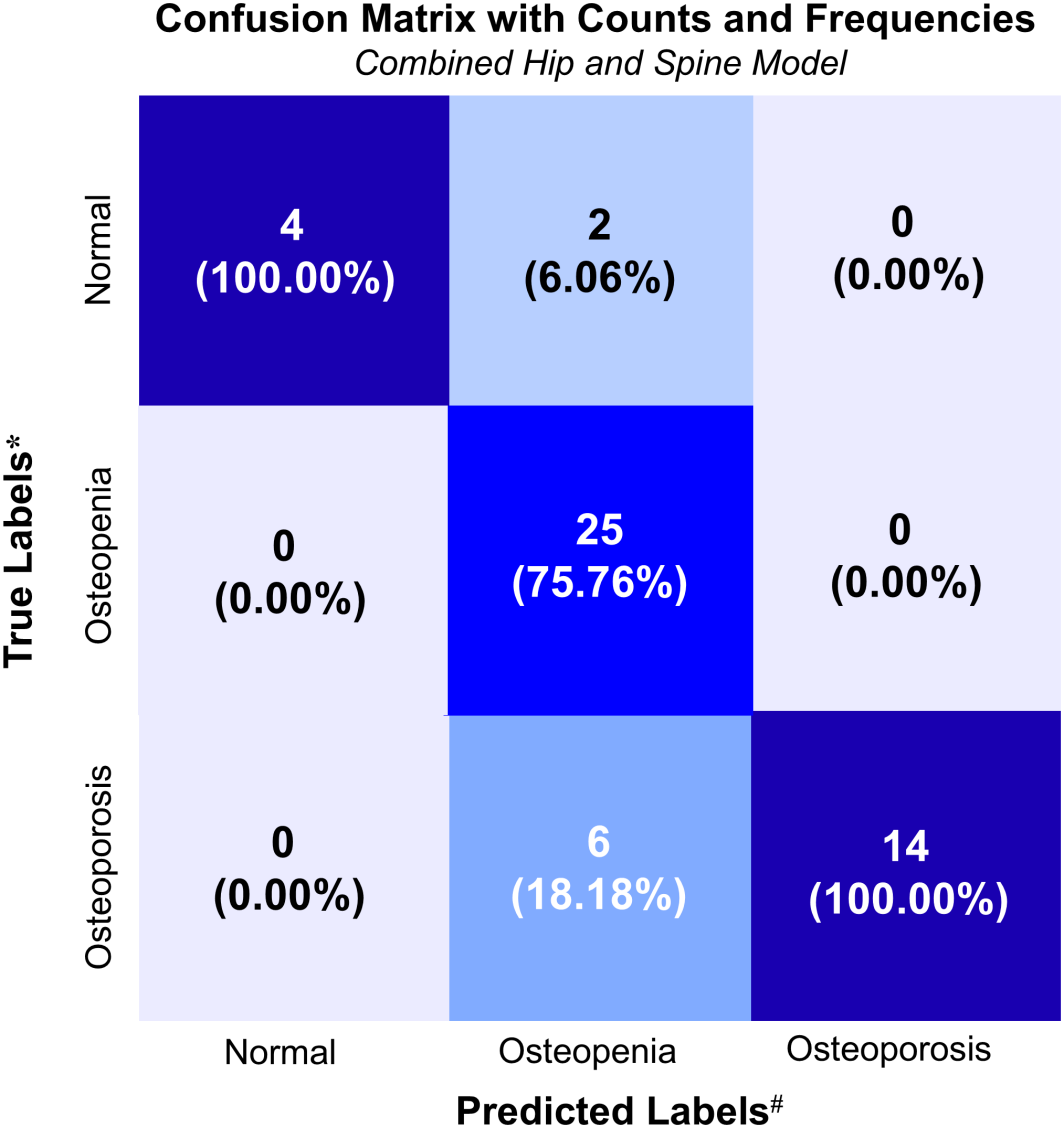
Confusion matrix of the combined spine and hip AI model in the prospective feasibility study. The matrix illustrates the diagnostic agreement between the AI model-predicted BMD categories and the ground truth DXA classification for the 51 participants. True diagnostic labels (derived from the lowest DXA T-score at the hip and spine) are displayed on the y-axis, and the AI-predicted labels (derived from the lowest model-predicted Tm-score) are on the x-axis. The values within each cell represent the absolute patient counts and their corresponding overall percentages. Using the conservative Tm-score threshold of ≤-2.8, the model effectively minimized false positives, achieving a 100% positive predictive value (PPV) and 100% specificity for osteoporosis prediction. BMD, bone mineral density; DXA, dual-energy X-ray absorptiometry.

Diagnostic evaluation using the optimized threshold of T_m_-score ≤-2.8 yielded a PPV of 100.0% (95% CI: 76.8-100.0), a specificity of 100.0% (88.8-100.0), and an AUC of 0.969 (0.924-1.000). Corresponding sensitivity and NPV were 70.0% and 83.8%, respectively, with zero false positives confirmed by the confusion matrix (**Figure 4**).

### Failure analysis

A retrospective review of false-negative cases—patients with DXA-confirmed osteoporosis but missed by the AI model at the ≤-2.8 threshold—revealed that the majority presented with significant spinal osteophytes or severe vascular calcifications. These anatomical abnormalities falsely elevated the radiomics-derived BMD predictions, highlighting an inherent limitation of 2D radiogrammetry in distinguishing cortical hypertrophy or superimposition from true trabecular bone density.

## Discussion

To the best of our knowledge, this is the first study to develop an AI algorithm for estimating dual-site BMD and facilitating osteoporosis screening using routinely acquired KUB radiographs. We developed BMD AI models, employing deep learning and radiogrammetric principles, which demonstrated excellent predictive performance both on the internal test set and, crucially, in the prospective feasibility study. The model provided accurate BMD estimations that were highly correlated with ground truth DXA measurements. Furthermore, by employing a conservative threshold optimized for opportunistic screening, the model achieved excellent PPV and specificity, confirming its potential utility for identifying osteoporosis cases from KUB radiographs while minimizing false positives. Our findings demonstrate that this radiogrammetry-based AI model can effectively estimate BMD, predict osteoporosis with robust performance, and show feasibility as an opportunistic osteoporosis screening tool in a real-world setting.

This study employed a deep-learning approach for model development, recognizing its strength in automatically discerning complex image patterns and subtle features that might escape human detection or traditional image analysis techniques (28, 29). Our AI models utilized a deep-learning framework (RegNetY-16GF backbone), leveraging the ability of deep neural networks to automatically extract complex, hierarchical features from image data. Recognizing the limitations of purely data-driven approaches, we incorporated several design elements to enhance performance and clinical relevance. For instance, an object detection model (YOLOv5) was fine-tuned to automatically segment the spine and hip ROIs (analogous to standard DXA), directing analysis to the area most associated with the highest risk of critical fracture, morbidity, and mortality. Furthermore, we integrated a hip landmark model (based on HRFormer) with established radiogrammetric analysis for precise anatomical feature extraction, identifying the most prominent LTR landmarks within the hip ROI to guide subsequent model training. This explicit integration of anatomical landmarking distinguishes our approach from models relying solely on convolutional feature extraction or texture analysis (30). The “feature-driven informed network for data refinement” step served as a vital quality control measure, ensuring only ROIs containing complete LTR and free from confounding factors were used for final BMD prediction. This refinement significantly improved model performance (**Supplementary Table 2**).

To fulfill the mandate of opportunistic screening —identify high-risk individuals while minimizing false positives—model optimization was deliberately targeted toward superior PPV and specificity (31, 32). Our exploration of modified T_m_-score thresholds identified a conservative T_m_-score ≤-2.8 as ab optimal cutoff, which yielded an excellent 100.0% PPV and 100.0% specificity in our prospective feasibility study. For opportunistic screening, where minimizing false alarms is critical, the inherent trade-off of lower sensitivity with a high-specificity approach is understood and accepted (33).

Compared to existing literature on AI applications in osteoporosis, our study’s use of KUB radiographs and the SHield pipeline represents an innovative approach. KUBs are frequently ordered in routine examinations and provide valuable information on both the spine and hip regions, critical sites for fragility fracture (34). The SHield pipeline integrates the predicted BMD from these dual sites to identify the lowest BMD value, aligning with clinical DXA reporting standards. This dual-site assessment from a single KUB broadens enhances the comprehensiveness of opportunistic screening, offering a distinct advantage over single-site methods (7, 10, 35). This is critical given the inherent inter-site heterogeneity of BMD—a common phenomenon in gold-standard DXA (27, 36). By evaluating both the spine and hip, the SHield pipeline accounts for these physiological variations, likely explaining its superior performance in identifying high-risk individuals compared to single-site AI models. Specifically, our models demonstrated high correlation with DXA BMD (*r* ≈ 0.89-0.96) and strong discriminatory ability for osteoporosis (AUC ≈ 0.89-0.97) reaching performance levels comparable to specialized, less-routine hip radiographs (15, 16, 18) and CT-based lumbar spine assessments (7, 37). Notably, our approach outperformed studies employing only chest X-ray (11) (AUC: 0.89), spine X-rays (12, 14) (AUCs: 0.73-0.81) or traditional X-ray radiogrammetry of the hand (10) (AUC: 0.76). Furthermore, by applying the optimized threshold specifically for opportunistic screening, the feasibility study achieved an exceptional PPV of 100.0%—a significant improvement over the 75.2% PPV reported in a recent randomized controlled trial using chest X-rays (11).

Building upon our model’s robust performance, it is noteworthy that some prior reports suggest that integrating clinical data can enhance the performance of deep learning models for osteoporosis prediction beyond using image alone. For instance, studies by Kim et al. (14) and Yamamoto et al. (16) demonstrated that adding clinical variables improved diagnostic performance over texture features or deep models alone. In contrast, our model achieved comparable high performance using deep features from KUBs, despite not incorporating clinical data. This underscores that KUBs contain sufficient diagnostic information for accurate assessment, independently providing a high-yield screening tool even when clinical metadata is unavailable.

There are some limitations to this study. First, the data refinement method’s requirement for a fully encompassed ROI and free from confounding factors led to the exclusion of a significant number of images, introducing potential selection bias, limiting the dataset size, and potentially narrowing its real-world applicability. However, this quality control measure contributed to the model’s high accuracy. Second, this sample size constraint also affected our prospective feasibility cohort (n=51). Consequently, performance metrics reaching 100% exhibited wide CIs when calculated using the exact binomial method. This reflects the statistical uncertainty inherent in small sample sizes, highlighting the need for future large-scale prospective studies to stabilize these estimates. Third, while this study utilized DXA ground truth from a single manufacturer (GE), BMD variations among different equipment brands present a potential challenge for generalization across institutions. Future study demonstrating model-estimated BMD values fall between those of the two most widely used manufacturers (GE and Hologic) would further validate its cross-brand capability. Lastly, the inherent “black box” nature of deep learning models presents a barrier to clinical trust and adoption (38). While the model performs well, incorporating explainability techniques in future work could improve transparency and clinical integration.

In conclusion, this study successfully developed and validated an AI pipeline capable of dual-site BMD and osteoporosis risk prediction from a single, existing KUB radiograph. This multi-site assessment approach aligns with the gold-standard DXA methodology, which requires at least two sites for an accurate diagnosis. By closely emulating this ground truth, our model demonstrated high accuracy for BMD estimation and excellent diagnostic performance in a prospective real-world setting. Ultimately, this approach offers a promising strategy to enhance early osteoporosis detection and potentially mitigate the global fracture burden.

## Data Availability

The original contributions presented in the study are included in the article/**Supplementary Material**. Further inquiries can be directed to the corresponding authors.
