# Supplementary material for: Comprehensive opportunistic osteoporosis screening with AI: retrospective development and prospective validation of dual-site BMD prediction from a single radiograph
Source: Front Endocrinol (Lausanne). 2026 Jul 23;17:1906679. doi: 10.3389/fendo.2026.1906679 (PMC13441782; doi:10.3389/fendo.2026.1906679)
Supplement: Supplementary file 1 [file Table1.docx]

**Supplemental Material of**

**Comprehensive Opportunistic Osteoporosis Screening with AI:**

**Retrospective Development and Prospective Validation of Dual-Site BMD Prediction from a Single Radiograph**

| Page 2 | Table S1: Study Sample Age Stratified by Sex in the prospective feasibility study |
| --- | --- |
| Page 3 | Table S2: Performance of the Hip Model in the Internal Test Set: Pre- and Post-FIND Method |

**Supplementary Tables**

**Table S1: Study Sample Age Stratified by Sex in the prospective feasibility study.***

| **Age** (year) | **Women** | **Men** | **Sum** |
| --- | --- | --- | --- |
| 50-59 | 21 (55.3%) | 17 (44.7%) | 38 |
| 60-69 | 20 (55.6%) | 16 (44.4%) | 36 |
| ≥70 | 18 (69.2%) | 8 (30.8%) | 26 |
| All | 59 (59.0%) | 41(41.0%) | 100 |

* Stratification was conducted to align the sex and age distribution of the study sample with the demographic profile of the Taiwanese population in 2022.

**Table S2: Performance* of the Hip Model in the Internal Test Set: Pre- and Post-FIND^†^ Method.**

| **MODEL** | **AUC**  (95%CI) | **PPV**  (%, 95%CI) | **NPV**  (%, 95%CI) | **Sensitivity**  (%, 95%CI) | **Specificity**  (%, 95%CI) | **RMSE** (95%CI) | **MAE** (95%CI) | **Pearson' r** (95%CI) | **OP prevale-nce^‡^** (%) |
| --- | --- | --- | --- | --- | --- | --- | --- | --- | --- |
| **Pre-**  **FIND^#^** | 0.877  (0.848-  0.903) | 75.6  (69.7-  81.5) | 82.7  (79.1-  86.0) | 69.1  (63.0-75.0) | 86.8  (83.7-90.1) | 0.076 (0.072-  0.080) | 0.059 (0.056-  0.062) | 0.841  (0.870-  0.902) | 33.9 |
| **Post-**  **FIND^#^** | 0.894  (0.868-  0.917) | 79.1  (73.2-  84.6) | 80.7  (77.1- 84.3) | 63.5  (57.3-70.0) | 90.1  (87.1-92.8) | 0.057  (0.055-  0.060) | 0.046 (0.044-  0.048) | 0.887  (0.870-0.902) | 29.8 |
| ***P*-value** | <0.001 | <0.001 | <0.001 | <0.001 | <0.001 | <0.001 | <0.001 | <0.001 | <0.001 |

* Osteoporosis ground truth was determined by DXA T-scores at hip and lumbar spine.

^†^ FIND: Feature-driven Informed Network for Data refinement.

^#^ A T_m_-score ≤ -2.5 was used as default detection threshold for osteoporosis (T_m_-score: T-scores derived from model-predicted

BMD values).

^‡^ OP prevalence was determined by applying the detection threshold to the T_m_-score.

BMD = bone mineral density, AUC = area under the receiver operating characteristic curve, PPV = positive predictive value, NPV = negative predictive value, RMSE = root mean squared error, MAE = mean absolute error, OP = osteoporosis, DXA = dual-energy X-ray absorptiometry.
